# Monitoring the effect of alcohol intake via facial temperature variations using thermography synchronized with heartbeat

**DOI:** 10.1038/s41598-025-17801-9

**Published:** 2025-09-01

**Authors:** Nanami Kotani, Kuniharu Sakurada, Jiayi Xu, Masahiko Inami, Yasuaki Monnai

**Affiliations:** Research Center for Advanced Science and Technology, The University of Tokyo, Tokyo, 153-8904 Japan

**Keywords:** Computer science, Information technology, Data integration, Data processing, Image processing, Time series, Signal processing

## Abstract

Accurate assessment of alcohol-induced physiological effects is critical for preventing overconsumption and ensuring safe activities such as driving. While breath alcohol concentration (BrAC) measurement is widely used due to its simplicity, it can be confounded by mouth alcohol effects and provides only momentary data. Building on our previously developed Synchro-thermography technique, which synchronizes infrared thermal imaging with heart rate variability to detect vascular-related skin temperature fluctuations, we applied it to monitor physiological changes induced by alcohol. In an alcohol intervention experiment, we observed that under non-drinking conditions, facial temperature fluctuations strongly synchronized with heartbeats. In contrast, following alcohol consumption, this synchrony markedly weakened within 10–30 minutes, regardless of the presence or absence of flushing responses. These results suggest that dynamic monitoring of temperature-heart rate synchrony offers a sensitive, non-contact indicator of early alcohol effects. Although preliminary, our findings highlight the potential of Synchro-thermography as a practical tool for non-invasive, real-time monitoring of alcohol-induced physiological changes.

## Introduction

Physiological changes following alcohol consumption, particularly during the hangover state, exert wide-ranging impacts on cognitive functions and daily activities. Previous studies have shown that heavy nighttime drinking substantially impairs delayed recall performance on the following morning, indicating that memory recovery processes are suppressed during hangover^1^. Other studies have similarly reported overall cognitive impairment during hangover^2^, which not only reduces individual productivity but also poses public safety risks. For instance, studies investigating post-drinking driving behavior have demonstrated that even when blood alcohol concentration (BAC) falls below legal thresholds, drivers tend to exhibit higher maximum speeds, longer periods of speeding, and greater variability in speed^3^. Moreover, individuals with alcohol flushing syndrome characterized by facial flushing, nausea, headache, and palpitations after consuming small amounts of alcohol^4^ require additional caution regarding drinking speed and quantity. Thus, to ensure driving safety and public health, it is critical to develop objective methods to assess whether residual alcohol-related effects persist in the body and to what extent.

Current methods for detecting alcohol effects primarily rely on the measurement of BAC or breath alcohol concentration (BrAC). While BAC measurements offer high accuracy, they are invasive and costly. BrAC measurements, although non-invasive and easy to perform, are susceptible to the mouth alcohol effect and capture alcohol levels only at discrete time points^5,6^. The Widmark formula^7^, widely used in forensic sciences to estimate BAC based on alcohol intake, elapsed time, gender, and body weight, also suffers from limited accuracy due to individual variability in alcohol metabolism and physiological parameters.

In recent years, infrared thermography has garnered attention as a non-contact, non-invasive technique for monitoring physiological states. Among them, the effect of blood perfusion on skin temperature is substantial. Therefore, numerous studies have focused on time series analysis of sequential thermal images to analyze hemodynamic effects^8–11^. In particular, it is well established that alcohol consumption can induce vascular dilation and elevate skin temperature^12,13^. Building on these findings, Koukiou et al.^14–17^ proposed thermal imaging methods to detect drinking behavior, achieving classification accuracies as high as 99 % based on facial vascular activity and scleral temperature increases^17^. However, these studies primarily focused on binary classification (i.e., drinker vs. non-drinker) and have yet to investigate the temporal dynamics of residual physiological states following alcohol intake.

To bridge this gap, the present study proposes an improved application of Synchro-thermography, a technique we recently developed, to monitor microvascular activity continuously and dynamically after alcohol consumption^18^. Synchro-thermography enhances conventional thermal imaging by synchronizing it with heart rate variability (HRV) signals, thereby stabilizing the detection of minute skin temperature fluctuations related to blood perfusion, even in facial regions where superficial vessels are sparse. In our prior work, we demonstrated the ability to detect subtle temperature changes (10 mK) with more than twofold improvement in thermal resolution, offering a promising platform for high-sensitivity vascular monitoring.

**Fig. 1:**
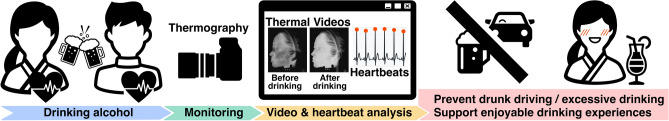
Graphical abstract. A schematic illustration of the research concept, methodology, and future applications of synchronized thermal imaging with heart rate variability for alcohol effect monitoring.

This study extends this approach to evaluate residual physiological changes associated with alcohol intake. Our proposed method features non-contact operation, high temporal resolution, and enhanced sensitivity, making it a promising candidate for objectively monitoring post-drinking cognitive and physiological status, driving fitness, and broader public health risk management. An overview of the research concept and potential applications is illustrated in Fig. 1. By synchronizing infrared thermal imaging with heart rate variability signals, this method enables sensitive, non-invasive detection of early alcohol-induced physiological alterations, offering a new technological alternative to conventional alcohol monitoring methods.

The following sections describe the experimental design, data acquisition procedures, and analysis methods used to validate the feasibility of this approach.

## Methods

This study conducted an alcohol intervention experiment to measure and analyze physiological, cognitive, and emotional changes following alcohol consumption using Synchro-thermography. The main purpose of this experiment is to verify the concept of the proposed method. Participants underwent a baseline resting-state measurement, a non-alcoholic beverage control condition, and an alcohol intervention condition, during which synchronized thermal imaging, visible video, ECG recording, alcohol sensitivity classification (flushing response questionnaire), cognitive assessment (Digit Span task), and emotional assessment (emoji questionnaire) were performed. The following sections provide a detailed description of the participants, experimental environment, measurement apparatus, experimental procedures, and analysis methods.

### Participants

Eight healthy East Asian participants (5 males and 3 females) took part in this study. The mean age of the participants was 25.5 years (SD 2.8 years). Ethical approval for the study was obtained from the Research Ethics Committee of the University of Tokyo (Approval No. 23-466), where the study was conducted. All experimental procedures and methods followed relevant ethical guidelines and regulations, including the Declaration of Helsinki. Participants were notified in advance that the study would involve alcohol consumption and were fully informed about the nature, procedures, and potential risks of the study. Written informed consent was obtained from all participants before the experiment. Participants were compensated for their time with an Amazon gift card (approximately 20 USD). In addition, written informed consent was obtained from the participants whose identifiable information or images appear in this article, allowing their use in an online open-access publication.

### Equipment and environment

**Fig. 2:**
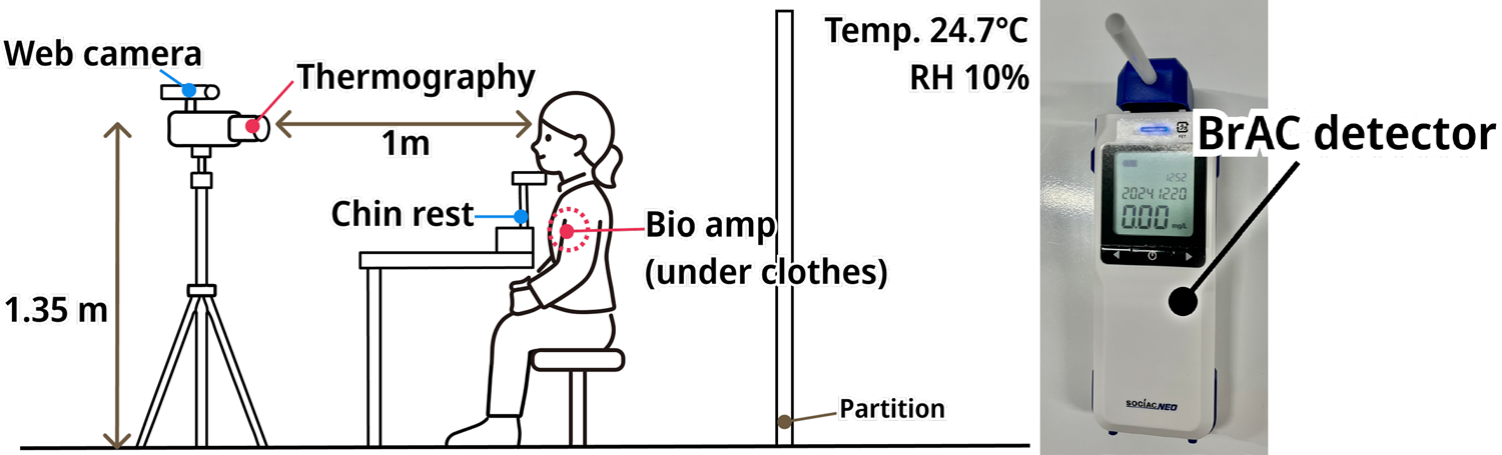
Experimental environment and Breath alcohol concentration detector used.

The experimental equipment and environment are shown in Fig. 2. Experiments were conducted in a room maintained at an average temperature of ${24.6}\,^\circ {\hbox {C}}$ (SD ${0.1}\,^\circ {\hbox {C}}$) and an average relative humidity of 10 % with air conditioning. The room size is ${6.2}\,{\hbox {m}} \times {8.0}\,{\hbox {m}}$ and the ceiling height is 3.7 m. The distance between the air conditioner and the subjects was sufficiently far apart so that the air conditioner breeze did not directly hit the subjects. Room temperature and humidity were constantly monitored by a temperature and humidity sensor placed at the same height as the thermography (1.35 m from the floor). The curtains were closed to prevent direct sunlight from entering the room.

To measure changes in facial temperature, a FLIR T530 thermal camera (FLIR Systems Inc.) was used. The thermal imaging system had a display resolution of $480 \times 640$ pixels and operated at a frame rate of 30 fps. Its temperature resolution (noise equivalent temperature difference) at ${30}\,^\circ {\hbox {C}}$ was less than 40 mK. Its absolute measurement accuracy is $\pm {2}\,^\circ {\hbox {C}}$. To ensure accurate thermal measurements, the thermography was configured with the following measurement parameters: target distance of 1 m, ambient temperature of ${25}\,^\circ {\hbox {C}}$, relative humidity of 10 %, reflected temperature of ${25}\,^\circ {\hbox {C}}$, and emissivity set to 0.98. The thermography was placed at a height of 135 cm from the floor and a distance of 100 cm from the participant. A partition was positioned 2.7 m in front of the thermal imaging camera to stabilize the background environment. In addition, a Logicool C922n webcam (Logitech Co., Ltd.) was mounted on top of the thermal camera to simultaneously capture both visible and thermal videos. To monitor participants heart activity, three electrodes were attached to the chest, and electrocardiogram (ECG) signals were recorded using a biometric amplifier (AMP-151, ATR-Promotions Inc.). The measurement range of the biometric amplifier is $\pm {0.4}\,{\hbox {mV}}$ to $\pm {4.8}\,{\hbox {mV}}$, and the specification is to amplify the measured value by 500 times. The ECG sampling rate was set at 200 Hz. Thermal imaging and ECG data acquisition were controlled by a single computer. Timestamps were recorded at the time of measurement and subsequently used for synchronization during data analysis. To measure participants’ blood alcohol concentration after drinking, breath alcohol concentration was assessed using a SOCIAC NEO SC-502 device (Central Automotive Products Ltd.), with participants exhaling into a straw. The SOCIAC series is one of the breath alcohol analyzers widely used by Japanese police for sobriety checkpoints and is known for its high accuracy and sensitivity.

### Experimental protocol

The experimental protocol was designed to systematically capture physiological, cognitive, and emotional changes associated with alcohol consumption under controlled conditions. Participants completed sequential phases, including a baseline measurement, a non-alcoholic beverage control phase, and an alcoholic beverage intervention phase, with repeated data acquisition sessions in each phase. To prevent subjects from reacting to and metabolizing non-alcoholic and alcoholic beverages differently depending on their physical condition, all sessions were conducted on the same day. Appropriate breaks were taken between each session to account for subject fatigue.

#### Procedure

**Fig. 3:**
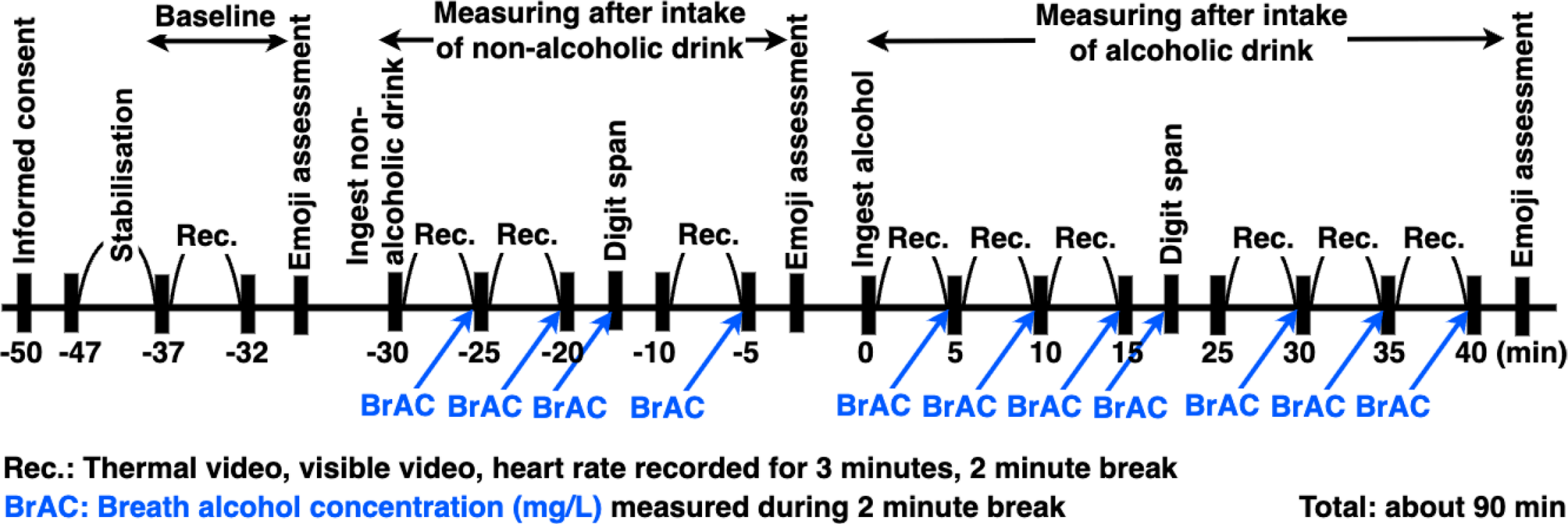
Experimental procedure and measurement schedule. Participants completed baseline, non-alcoholic beverage, and alcoholic beverage phases with repeated thermal imaging, ECG, cognitive, and emotional assessments.

The detailed experimental procedure is illustrated in Fig. 3. As shown in Fig. 3, one recording session consisted of 3 minutes of measurement and 2 minutes of rest, for a total of 5 minutes.

1. **Informed consent**: Participants completed the consent form and pre-participation questionnaire to ensure full understanding of the study content and potential risks. The pre-participation questionnaire also included items assessing alcohol flushing response, allowing participants to be categorized into flushing and non-flushing groups based on their self-reported sensitivity to alcohol metabolism.
2. **Preparation**: Electrodes for heart rate measurement were attached to the chest, and participants rested for 10 minutes to acclimate to the room temperature.
3. **Resting state measurement (1 time)**: Baseline physiological data were recorded. An emoji questionnaire (Table S2) was administered at the end of the session to assess emotional state.
4. **Measurements after consuming non-alcoholic beverages (3 times)**: After drinking a non-alcoholic beverage, physiological data were recorded three times. A Digit Span cognitive test was administered after the second measurement. An emoji questionnaire was administered after the third measurement.
5. **Measurements after consuming alcoholic beverages (6 times)**: After drinking an alcoholic beverage, physiological data were recorded six times. A Digit Span cognitive test was administered after the third measurement. An emoji questionnaire was administered after the sixth measurement.

Each measurement session included 3 minutes of thermal video, visual video, and ECG recording, followed by a breath alcohol concentration measurement. Participants sat with their heads placed on a chin rest in a comfortable and stable position during filming. Participants kept their hair out of their faces during the experiment. Recording was conducted from the left oblique side of the face, based on previous findings that there is no significant temperature asymmetry between the left and right sides of the face^18,19^.

#### Beverage preparation

To achieve consistent and controlled alcohol administration across participants, beverage preparation was carefully designed based on physiological modeling and previous studies.

In this experiment, alcohol was consumed to achieve a blood alcohol concentration (BAC) of approximately 0.05 %. The alcohol used was 80 proof vodka (alcohol strength 40 %). Based on the Widmark method^7^, the volume of alcohol beverage required to reach a BAC of $C_t = {0.05}\,{\%} = {0.5}\,{\hbox {mg/mL}}$ was pre-calculated. Given that the density of ethanol is ${0.8020}\,{\hbox {g/cm}^{3}}$ at ${5}\,^\circ {\hbox {C}}$ and ${0.7978}\,{\hbox {g/cm}^{3}}$ at ${10}\,^\circ {\hbox {C}}$^20^, the density $d = {0.8}\,{\hbox {g/cm}^{3}}$ was used in the preliminary calculations. Assuming an alcohol strength $z = 0.4$, elapsed time $t = {0}\,{\hbox {s}}$, and body weight *M* kg, the Widmark formula^7^ gives:

1 $$\begin{aligned} 0.5&= \frac{vzd}{rM} - \beta t = \frac{0.4 \cdot 0.8 v}{rM} \nonumber \\ v&= 1.5625 \cdot rM \end{aligned}$$

where *r* is the volume of distribution of ethanol, set as $r = 0.7$ for males and $r = 0.6$ for females^21^. Thus, the final amount of alcohol consumed per 1 kg of body weight was 1.09 mL/kg for males and 0.938 mL/kg for females.

Beverages were prepared following previous research on alcohol administration protocols^22–24^. Non-alcoholic beverages were mixed at a ratio of cranberry juice : lime juice : tonic water $= 1:1:2$, while alcoholic beverages were prepared at a ratio of cranberry juice : lime juice : tonic water : vodka (80 proof) $= 1:1:1:1$. To limit individual differences in total beverage volume based on body weight and gender, the total volume of each drink was adjusted to be less than or equal to 200 mL. For example, for a male participant weighing 70 kg, the calculated vodka volume was 76.6 mL. Without adjustment, the total beverage volume would be ${76.6}\,{\hbox {mL}} \times 4 = {306.4}\,{\hbox {mL}}$. Thus, the non-alcoholic components (cranberry juice, lime juice, tonic water) were each reduced to approximately $(200-76.6)/3 \approx {41.1}\,{\hbox {mL}}$.

All beverages were stored in cooler boxes throughout the experiment. The average temperature for non-alcoholic beverages was ${6.2}\,^\circ {\hbox {C}}$ (SD ${1.0}\,^\circ {\hbox {C}}$) and for alcoholic beverages was ${9.6}\,^\circ {\hbox {C}}$ (SD ${1.0}\,^\circ {\hbox {C}}$). Non-alcoholic beverages were consumed first, followed by alcoholic beverages. Although beverages were kept cool, the temperature of alcoholic beverages slightly increased due to the order of consumption.

#### Flushing response classification

The alcohol flushing questionnaire was used to classify participants based on their sensitivity to alcohol metabolism, which may influence physiological responses following drinking. Participants answered two questions regarding their tendency to experience facial flushing after alcohol intake^4^. Those answering “yes” to either question were classified into the flushing response group, and those answering “no” to both were classified into the non-flushing group.

1. Do you currently tend to get red in the face after drinking a small amount of alcohol, such as a glass of beer? (yes/no)
2. Did you tend to flush immediately after drinking a glass of beer in the first to second year after you started drinking? (yes/no)

#### Digit span test

The Digit Span task was used to assess working memory performance before and after alcohol consumption, allowing investigation of potential cognitive impairments associated with drinking. Digit Span (Digits Forward and Digits Backward) was conducted online using computers in the specified order^25^, following the protocol of the Wechsler Adult Intelligence Scale (WAIS)^26–28^. The maximum number of digits correctly recalled was recorded as the score.

#### Emoji questionnaire

The emoji questionnaire (Table S2) was used to non-verbally measure emotional changes before and after drinking, independent of participants’ native language. The questionnaire was adapted from a previous study^29^, consisting of 30 selected emojis based on valence and arousal ratings. Three emojis with overlapping emotional values were excluded to simplify the analysis. Higher positions corresponded to higher arousal levels, and the horizontal axis represented valence from negative (left) to positive (right).

### Thermal video analysis

**Fig. 4:**
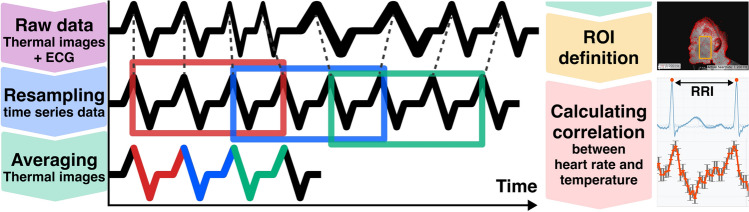
Synchro-thermography analysis pipeline. Thermal images were synchronized with ECG-derived heartbeats, resampled, averaged, and cross-correlated to evaluate dynamic skin temperature fluctuations associated with cardiac activity.

This section describes the analysis procedure using Synchro-thermography, a technique developed to detect minute skin temperature fluctuations synchronized with heart rate variability (HRV). Since blood perfusion dynamics are closely influenced by cardiac cycles, synchronizing thermal measurements with heartbeats enables the sensitive and stable detection of vascular-related temperature changes. By enhancing the signal-to-noise ratio through heartbeat-synchronized averaging, Synchro-thermography provides a robust means of monitoring subtle physiological changes non-invasively. The detailed analysis pipeline is illustrated in Fig. 4.

1. **Preprocessing:** R-peaks were detected from ECG signals using Christov’s algorithm^30,31^.
2. **Resampling:** The heartbeat signals and thermal video frames were resampled into equally spaced time series data based on the measured mean RR interval. Subsequently, RIPOC^32^ was applied to align the time-scaled thermal images and compensate for minor subject movements.
3. **Synchronous averaging:** A sliding-window method was employed to perform synchronous additive averaging over the aligned thermal images. The averaged images were concatenated to construct a new thermal video dataset.
4. **Region of interest (ROI) definition:** A rectangular region encompassing the cheeks and chin—areas easily and consistently set across all participants—was manually defined as the region of interest (ROI). The ROI was visually confirmed to be an exposed area with no hair and not covered with eyes, nose, or mouth.
5. **Calculating correlation:** To assess the relationship between skin temperature variation and heart rate variability, cross-correlation analysis was performed between the skin temperature signals in the ROI and a reference cosine wave derived from the average RR interval. The correlation coefficients were computed by shifting the central pixel of each 25-pixel local region by one pixel at a time within the ROI. The resulting coefficients were then ranked by their absolute values.

For each 3-minute scan, the 30-second segment with the least body motion was selected for analysis. The sliding-window parameters were set to a window size of 10 heartbeats and a step size of 3 heartbeats, adjusted according to each participant’s heartbeat interval. Skin temperatures and standard errors were calculated as in our previous study^18^.In brief, skin temperature measurements were calculated by averaging the value of a central pixel (*x*, *y*) and the surrounding 24 pixels from $(x-2, y-2)$ to $(x+2, y+2)$, totaling 25 pixels. Our prior study^18^ qualitatively suggested a correlation between body temperature fluctuation and heart rate using the proposed method. To quantitatively assess this relationship, we used the Pearson correlation coefficient (Eq. (2)). This metric was chosen due to the presence of very few outliers in body temperature and heart rate values and its suitability for exploratory trend analysis. Since the data are discrete real-domain values, the Pearson correlation coefficient was computed by Eq. (2) where *x*, *y* represent body temperature and heart rate, and lag *k* is an integer between 0 and the RR interval.

2 $$\begin{aligned} r_{xy}(k)&= \Sigma _{i=1}^{n-k} (x_{i+k} \cdot y_i) / \sqrt{\Sigma _{i=1}^n x_i^2 \Sigma _{i=1}^n y _i^2} \end{aligned}$$

When ranking correlation values, nearby coordinates whose x- or y-distance differed by less than 10 pixels from higher-ranked points were excluded to avoid spatial redundancy.

## Results

Following the experimental procedures described above, we analyzed the collected data focusing on four aspects: (1) alcohol sensitivity classification based on flushing response, (2) physiological responses, including changes in body temperature and breath alcohol concentration after beverage ingestion, (3) cognitive performance assessed using the Digit Span task, and (4) emotional state variations evaluated through the emoji questionnaire. The detailed results for each aspect are reported below.

### Alcohol sensitivity classification

Based on responses to the alcohol flushing questionnaire, the 8 participants were classified into two groups: 4 (2 males, 2 females) in the flushing group and 4 (3 males, 1 female) in the non-flushing group. In subsequent analyses, comparisons between the flushing and non-flushing groups were performed for physiological, cognitive, and emotional responses.

### Physiological response to beverage ingestion

#### Changes in body temperature and breath alcohol concentration

**Fig. 5:**
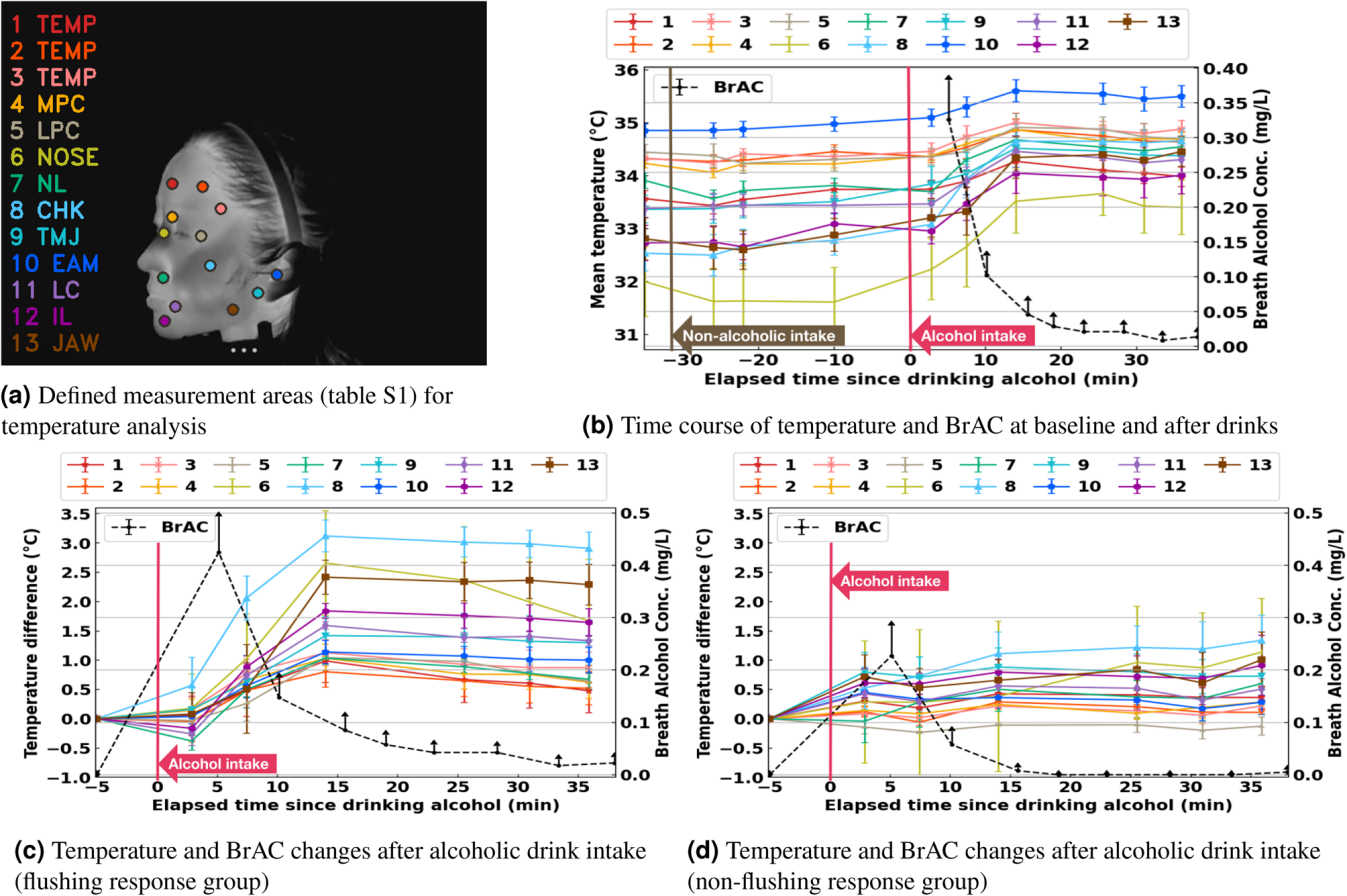
Temperature responses at the defined measurement areas (**a**) and breath alcohol concentration (BrAC) following beverage ingestion. **b** Time course of temperature and BrAC at baseline, after intake of a non-alcoholic beverage, and after intake of an alcoholic beverage. **c** and **d** show the corresponding changes separately for the flushing response group and the non-flushing response group. After obtaining the temperatures averaged over 30-second windows for each individual, they were used to calculate the mean temperature and standard error for each group. BrAC was 0 mg/L in all subjects before alcohol intake. Error bars indicate the standard error of the mean (s.e.m.).

To eliminate the influence of beverage temperature on skin temperature measurements and accurately assess physiological changes induced by alcohol consumption, we expanded the body surface measurement areas based on the setup by Haddad et al.^19^, establishing 13 temperature measurement points as shown in Table S1 for a more detailed and comprehensive observation of temperature variation characteristics. An actual example of the measurement points shown in Table S1 is shown in Fig. 5a. These measurement points were set manually on the GUI to set the same corresponding position for each subject.

Figure 5 presents changes in body temperature and breath alcohol concentration (BrAC) under three conditions: baseline (resting), after intake of a cold non-alcoholic drink, and after intake of a cold alcoholic drink. Temperature and BrAC are shown as group averages for the flushing and non-flushing groups, reflecting overall physiological trends rather than individual cases.

As shown in Fig. 5b, body temperature remained largely stable after ingestion of a cold non-alcoholic beverage, whereas it increased markedly after alcoholic intake, suggesting that the changes were driven by alcohol-induced physiological responses rather than beverage temperature. Further analysis of group differences revealed that in the flushing group, body temperature peaked approximately 15 minutes after drinking (Fig. 5c), whereas in the non-flushing group, slight increases were observed in regions such as the cheeks and temples (Fig. 5d). This trend is consistent with previous findings^13^, which reported that initial physiological responses to alcohol generally occur within 20 minutes.

Regarding BrAC (black line in the figures), concentrations rose rapidly after drinking. They declined to nearly 0 mg/L within approximately 15 minutes, suggesting that early BrAC measurements were mainly influenced by residual alcohol in the oral cavity. Notably, BrAC decreased more slowly in the flushing group, indicating possible individual differences in alcohol metabolism or clearance capacity. Around 35 minutes after drinking, BrAC levels rose again in all participants, implying the true onset of elevated blood alcohol concentration and systemic metabolism.

In summary, due to the confounding effect of oral alcohol residue, relying solely on early post-drinking BrAC measurements cannot accurately reflect the true blood alcohol level. Furthermore, although body temperature increases were more prominent in the flushing group, the magnitude of change in the non-flushing group was relatively small. Therefore, facial thermal imaging alone is insufficient for universally assessing individual alcohol effects, highlighting the need for combined analyses using dynamic physiological indicators with higher temporal resolution.

#### Correlation between skin temperature fluctuation and heart rate variability

To further investigate the impact of alcohol ingestion on physiological synchrony, we analyzed the correlation between skin temperature fluctuations and heart rate variability (HRV) based on synchronized thermal imaging data.

The detailed methodology is described in the *Methods* section: R-peaks were detected from ECG signals using Christov’s algorithm, and both ECG and thermal videos were resampled. A sliding-window synchronous averaging method was employed to generate smoothed temporal sequences. Within the defined regions of interest (ROIs), Pearson correlation analysis was performed between the skin temperature signals and a cosine reference wave constructed based on the average RR intervals to evaluate the degree of synchrony between temperature and heart rate variability.

Table 1 summarizes the average absolute correlation coefficients of the top 10 pixels between skin temperature and heart rate for each measurement phase. Non-alc 1–3 correspond to the sequential measurements after non-alcoholic beverage intake, and Alc 1–6 correspond to those after alcoholic beverage intake. Overall, temperature-heart rate correlations remained moderate ($0.4 < |r| \le 0.6$) during baseline and after non-alcoholic beverage ingestion, but dropped to weak correlation levels ($|r| \le 0.4$) approximately 14 minutes after alcohol ingestion. The correlation strength classification in this study is as follows:

- Strong correlation: $$|r| > 0.6$$
- Moderate correlation: $$0.4 < |r| \le 0.6$$
- Weak correlation: $$|r| \le 0.4$$

Regardless of flushing response, correlations between temperature and heart rate variability decreased notably about 14 minutes after alcohol intake, suggesting that alcohol disrupts the rhythmic perfusion dynamics. In the flushing group, the desynchronization persisted longer, whereas in the non-flushing group, partial recovery was observed over time.

**Table 1:** Average absolute correlation coefficients of the top 10 pixels between skin temperature and heart rate under each condition. “Non-alc” represents the post-intake of a non-alcoholic drink, and “Alc” represents the post-intake of an alcoholic drink. The numbers indicate sequential measurement sessions. The mean (standard deviation) is written.

	Baseline	Non-alc 1	Non-alc 2	Non-alc 3	Alc 1	Alc 2	Alc 3	Alc 4	Alc 5	Alc 6
Flushing: Mean	0.53	0.56	0.54	0.60	0.57	0.50	0.37	0.29	0.31	0.32
SD	0.07	0.07	0.14	0.13	0.12	0.14	0.15	0.14	0.15	0.09
Non-flushing: Mean	0.51	0.57	0.43	0.53	0.38	0.48	0.38	0.48	0.45	0.48
SD	0.10	0.07	0.11	0.12	0.17	0.17	0.09	0.11	0.03	0.15
Overall average: Mean	0.52	0.56	0.48	0.56	0.48	0.49	0.38	0.38	0.38	0.40
SD	0.08	0.06	0.13	0.13	0.17	0.14	0.11	0.16	0.12	0.14

**Fig. 6:**
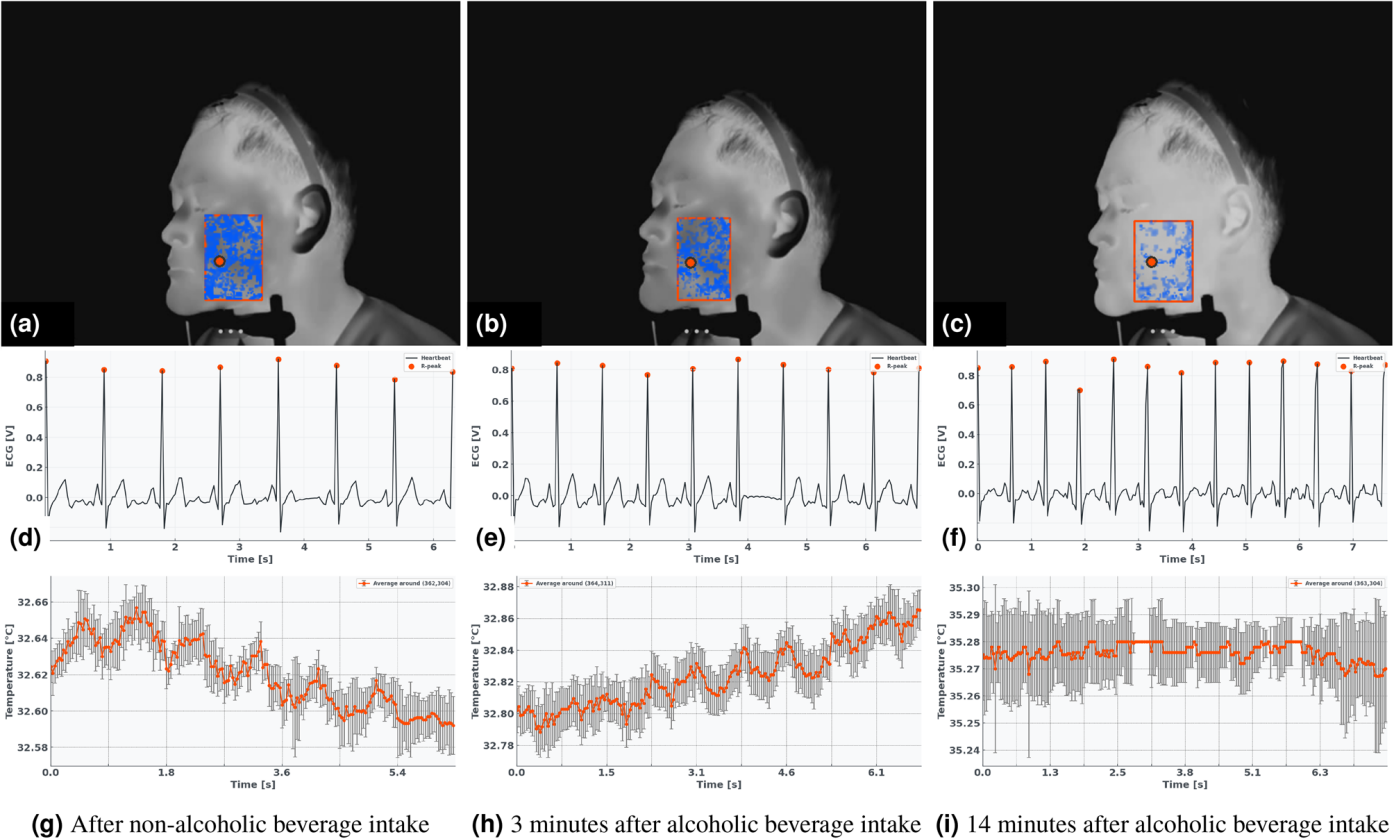
Changes in the correlation between heart rate and facial temperature in a subject with a flushing response following beverage ingestion. **a**–**c** Thermal images where the blue-colored areas indicate regions with stronger absolute correlations between heart rate and facial temperature. The red box denotes the region of interest (ROI), and the temperature fluctuation at the red point is plotted in (**g**)–(**i**). The red points represent the top 10 pixels with the highest correlation coefficients. **d**–**f** Resampled heartbeat waveforms corresponding to each condition. Using the proposed method, the correlations from the raw data were improved as follows: 0.17 to 0.64 for (**g**), 0.08 to 0.59 for (**h**), and 0.12 to -0.38 for (**i**). Error bars represent the standard error of the mean (s.e.m.).

Figure 6 shows an example from a participant in the flushing group. Following alcohol ingestion, the areas showing high correlation between heart rate and temperature (depicted in blue) progressively decreased compared to the state after non-alcoholic beverage ingestion (Fig. 6a–c). Furthermore, tracking the point with the highest correlation revealed that its temperature increased from approximately ${32}\,^\circ {\hbox {C}}$ to ${35}\,^\circ {\hbox {C}}$, while its cyclic correlation value dropped from 0.64 to – 0.38, indicating substantial desynchronization.

**Fig. 7:**
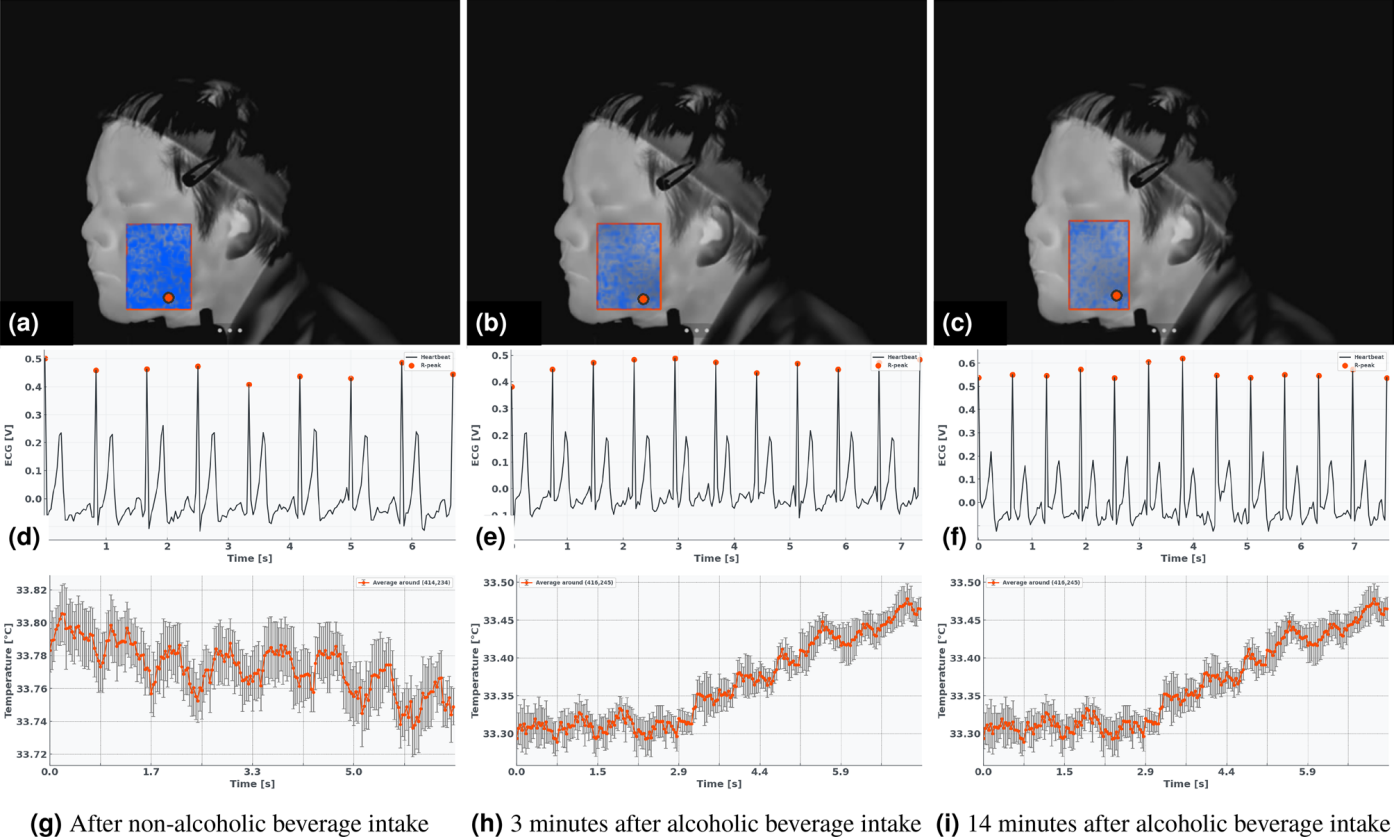
Changes in the correlation between heart rate and facial temperature in a subject without a flushing response following beverage ingestion. **a**–**c** display thermal images where the blue-colored areas indicate regions with stronger absolute correlations between heart rate and facial temperature. The temperature fluctuation at the red point is plotted in (**g**)–(**i**). **d**–**f** Resampled heartbeat waveforms corresponding to each condition. Using the proposed method, the correlations from the raw data were improved as follows: 0.10 to – 0.65 for (**g**), –0.11 to 0.34 for (**h**), and 0.02 to –0.23 for (**i**). Error bars represent the standard error of the mean (s.e.m.).

Figure 7 presents a non-flushing group example. Although the overall temperature rise was less pronounced, the correlation coefficient of the highest-correlated pixel also declined from -0.65 to -0.23 after alcohol ingestion, suggesting that physiological synchrony disruption occurs even in the absence of apparent skin temperature elevation.

Figure S1 shows the evolution of the correlation coefficient between body temperature and heart rate for subjects with a flushing response, shown in Fig. 6, and subjects without a flushing response, shown in Fig. 7. It can be seen that alcohol consumption lowers the maximum of the correlation coefficient. In addition to the maximum value of the correlation coefficient, the decrease in the area of high correlation coefficient from Figs. 6 and 7 also suggests that the correlation coefficient may be a measure of intoxication.

Therefore, these results indicate that alcohol consumption impairs the synchrony between skin temperature fluctuations and cardiac activity. This loss of physiological rhythmicity may reflect changes in perfusion regulation and highlights the potential of using dynamic synchrony metrics as more stable physiological markers for assessing individual responses to alcohol.

### Cognitive performance: digit span task

Digit Span tasks (forward and backward) were used to evaluate working memory capacity before and after alcohol ingestion. No considerable decline in Digit Span scores was observed immediately after alcohol ingestion across all participants. The mean Digits Forward score before drinking was 8.1 (SD = 0.83), and Digits Backward was 6.6 (SD = 1.1). After drinking, the scores were 7.6 (SD = 1.6) and 8.0 (SD = 1.3), respectively.

Although slight individual variations existed, no clear difference was observed between the flushing response group and the non-flushing response group. Due to the small sample size, statistical testing was not performed. Thus, while physiological changes were apparent, immediate cognitive impairments were not pronounced under the moderate alcohol load used, regardless of alcohol sensitivity.

### Emotional state variations: emoji questionnaire

Emotional states were assessed using the emoji questionnaire administered before and after beverage ingestion. The most common emoji overall at baseline was  (neutral), answered by 6 participants (3 in the flushing group and 3 in the non-flushing group). The most common response in both groups, with and without flushing reaction, was . After consuming the non-alcoholic drink, the most common emoji overall was  answered by 3 participants (3 in the non-flushing group). The most common response in the non-flushing group was also . The most common responses in the flushing group were  (tired),  (smiling),  (happy),  (sleepy), given by 2 participants. After alcohol consumption, the most common response overall was  (sleepy) answered by 6 participants (3 in the flushing response group and 3 in the non-flushing response group). The most common response in the non-flushing response group was  answered by 3 participants. On the other hand, the most common response in the flushing response group was  (blushing), which was reported by all four participants.

This result indicates that alcohol consumption induced drowsiness. In addition, the fact that the flushing response group all responded  indicates that they are aware that alcohol causes their facial temperature to rise and become red. Due to the limited sample size, these findings should be interpreted cautiously, but they suggest that moderate alcohol intake may subtly modulate emotional states in a participant-specific manner.

## Discussion

This study investigated physiological, cognitive, and emotional changes associated with alcohol consumption using synchronized thermal imaging with heart rate variability (Synchro-thermography). The key findings are summarized as follows.

Alcohol intake substantially disrupted the synchrony between facial skin temperature fluctuations and heart rate variability, irrespective of the presence or absence of a facial flushing response. A notable rise in facial skin temperature was observed approximately 15 minutes after alcohol ingestion among participants with a flushing response, whereas minimal changes in average skin temperature were detected in the non-flushing group. Importantly, conventional breath alcohol concentration (BrAC) measurements taken immediately after drinking were strongly influenced by residual alcohol in the oral cavity, potentially leading to underestimation of systemic alcohol levels. In contrast, Synchro-thermography promptly captured physiological changes associated with vascular activity, providing a more stable indicator less affected by oral artifacts. Furthermore, moderate alcohol consumption under the current experimental conditions did not result in substantial short-term impairment of working memory performance (Digit Span task). After drinking alcohol, the number of participants who answered  (sleepy) increased regardless of whether they had a flushing reaction. However, the sample size was insufficient to test the relationship between changes in emotional state (emoji questionnaire in table S2) and temperature.

These results suggest that dynamic monitoring of the synchrony between skin temperature and cardiac activity can serve as a sensitive and non-invasive indicator of early alcohol effects, potentially overcoming limitations associated with traditional BrAC measurements.

Despite these promising findings, several limitations should be acknowledged. First, the sample size was small and limited to East Asian participants. This restricts the generalizability of the findings. Future studies with larger and more ethnically diverse cohorts are needed to validate the robustness and cross-population applicability of Synchro-Thermography in alcohol-related physiological monitoring. Second, the monitoring window after alcohol ingestion was restricted to approximately 45 minutes to control experimental duration. Extending the observation period in future work would enable a more comprehensive assessment of the later stages of alcohol metabolism and recovery. Third, while it is hypothesized that alcohol-induced vasodilation contributed to the observed decrease in temperature heart rate synchrony^33,34^, direct physiological validation was not performed. Future studies should consider combining Synchro-thermography with vascular imaging techniques such as laser Doppler flowmetry or near-infrared spectroscopy to clarify the underlying hemodynamic mechanisms. Fourth, the region of interest (ROI) was manually selected in this study to account for individual differences in facial features and to enable more accurate comparisons of temperature changes associated with alcohol consumption. However, given the extensive research on facial feature detection in visible-spectrum images^35,36^, it is considered feasible to adapt these techniques to thermal images for the automatic selection of ROIs. Fifth, while the present study measured heart rate using chest-attached ECG electrodes, this method inherently requires physical contact and may limit the practicality of the system in real-world environments. A promising alternative is the adoption of remote photoplethysmography (rPPG), which estimates heart rate from visible-light facial video^37,38^. rPPG enables fully contactless monitoring, eliminating the need for electrodes and improving comfort, hygiene, and scalability. This technology aligns well with the core strength of Synchro-Thermography non-invasiveness and could facilitate truly remote, multi-subject physiological monitoring in contexts such as public intoxication screening, workplace safety, or in-vehicle driver monitoring. Integrating rPPG with thermal imaging would thus be an important step forward toward developing a practical, scalable, and fully contactless alcohol monitoring system.

Overall, compared to conventional methods, the combination of synchronized thermal imaging and heart rate variability analysis offers distinct advantages: it avoids false negatives due to oral alcohol residue, enhances sensitivity to subtle microvascular changes, enables dynamic and continuous monitoring with temporal resolution, and holds potential for early detection of excessive drinking or individualized assessment of alcohol metabolism. These findings highlight Synchro-thermography as a promising, non-invasive, and personalized approach for detecting early alcohol-induced physiological changes, paving the way for new strategies in intoxication detection, risk prediction, and real-time health monitoring.

## Supplementary Information

Supplementary Information.

## Data Availability

The anonymized datasets generated during this study can be made available from the corresponding author upon reasonable request. The raw thermal images of the subjects will not be available due to privacy regulations.

## Code availability

The code is available at https://github.com/NKotani/synchro-thermography.
