# Supplementary material for: Monitoring the effect of alcohol intake via facial temperature variations using thermography synchronized with heartbeat
Source: Sci Rep. 2025 Sep 1;15:32249. doi: 10.1038/s41598-025-17801-9 (PMC12402095; doi:10.1038/s41598-025-17801-9)
Supplement: Supplementary file 1 — Supplementary Information. [file 41598_2025_17801_MOESM1_ESM.pdf]

Table S1. Correspondence between measurement areas and anatomy

ROIs	Names of regions	Anatomical vascularity
ST	Supratrochlear	Supratrochlear artery
TEMP	Temporal	Superficial temporal artery
MPC	Medial palpebral commissure	Dorsal nasal artery, Angular artery
LPC	Lateral palpebral commissure	Zygomatico-orbital artery
NOSE	Nose	Dorsal nasal artery
NL	Nasolabial	Superior labial branch
CHK	cheek	Transverse facial artery
TMJ	Temporomandibular joint	Superficial temporal artery
EAM	External acoustic meatus	Posterior auricular artery
LC	Labial commissure	Facial artery
IL	Inferior labial	Inferior labial branch
JAW	jaw	Facial artery

Table S2. Emoji questionnaire

angry	confused	scared	laughing	crazy	love
exhausted	crying	awkward	embarrassed	smiling	happy
tired	helpless	smirk	silly	joke	kissing
bad	stressed	dissatisfied	neutral	grinning	blushing
disappointed	sad	unhappy	indifferent	sleepy	relief

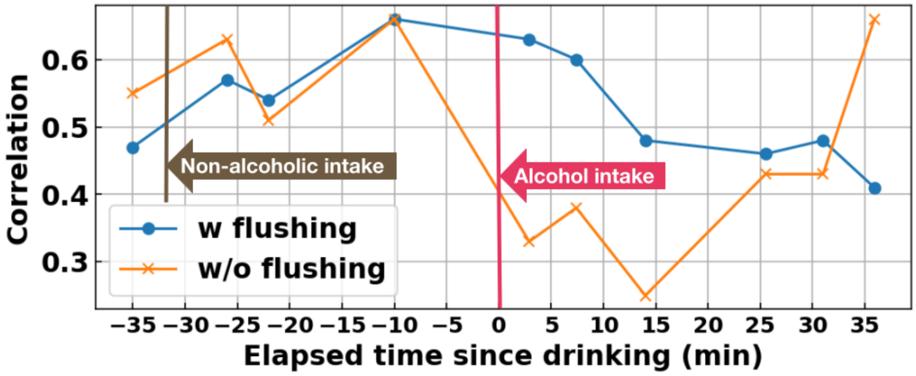

Figure S1. Correlation coefficients between facial temperature and heart rate variability in participants with flushing response (Figure 6) and without flushing response (Figure 7)
